# Post-traumatic stress disorder in a national sample of preadolescent children: Prevalence, correlates, clinical sequelae, and treatment utilization

**DOI:** 10.21203/rs.3.rs-3303568/v1

**Published:** 2023-11-15

**Authors:** Rachel Levin, Richard Liu

**Affiliations:** University of Rochester; Harvard Medical School

## Abstract

Although posttraumatic stress disorder (PTSD) has been well characterized in adults, its epidemiology in children is unclear. The current study provides the first population-based examination of the prevalence of PTSD, sociodemographic and psychiatric correlates, clinical sequelae, and associations with psychiatric treatment in preadolescents in the United States. Data from the Adolescent Brain and Cognitive Development (ABCD) Study (release 5.0) was analyzed. Participants (unweighted *n* = 11, 875) were recruited from 21 sites across the United States. Current and lifetime PTSD prevalence were estimated, as was treatment use among children with PTSD. Sociodemographic, psychiatric correlates and sequelae of PTSD were analyzed using logistic regression, as was the association between PTSD and psychiatric treatment. Lifetime prevalence of PTSD was 2.17%. Sexual minority status, being multiracial, having unmarried parents, and family economic insecurity were associated with greater odds of PTSD. Among psychiatric disorders, separation anxiety was most strongly associated with PTSD, although general comorbid psychopathology was associated with greater odds of PTSD. Prior history of PTSD predicted new onset of other psychiatric disorders after PTSD remission. Nearly one in three children with lifetime PTSD did not receive psychiatric treatment, despite negative long-term outcomes of PTSD and significant psychiatric comorbidity. Even among preadolescents who experience full remission of PTSD, significant risk for future psychiatric illness remains. Further, the current findings underscore the need for improved efforts to reduce unmet treatment needs among those with PTSD at this age.

## Introduction

Post-traumatic stress disorder (PTSD) has been well characterized in adults. In the United States, national studies have found lifetime PTSD prevalence of 7.3%^1^ and 7.8%.^2^ In the one epidemiological study to date to examine this issue in adolescents, a lifetime prevalence of 4.7% was found.^3^

Although PTSD has been widely reported to occur in younger children, the epidemiology of this disorder in preadolescents remains unclear. Indeed, most prior studies with this age group have featured very small samples,^4^ which tend to yield unstable prevalence estimates and limit generalizability of their findings. In perhaps the largest study to date of PTSD in a population-representative sample of preadolescent children, the lifetime prevalence was 0.1%.^5^ Although based on a large community sample, these findings are representative of a regional population only and cannot be generalized to the national population. Furthermore, the small number of cases of PTSD precluded any determinations of associated sociodemographic factors, diagnostic correlates, and prevalence of psychiatric care. While research in adolescents and young adults has shown the development of comorbid psychopathology after PTSD diagnosis,^6^ the clinical sequelae of PTSD in preadolescents is unknown. Being able accurately to characterize PTSD and evaluate its clinical sequelae in this age group is especially important for understanding the development of this disorder and to inform prevention and intervention services.

The current study aimed to provide the first comprehensive assessment of PTSD in a population-based sample of preadolescent children. Specifically, its objectives were: (i) to estimate the lifetime prevalence of preadolescent PTSD in the general population; (ii) to assess sociodemographic and psychiatric correlates of this disorder; (iii) to evaluate PTSD as a predictor of subsequent onset of new psychopathology; (iv) to generate estimates of the prevalence of lifetime psychiatric treatment among preadolescents with PTSD, thereby providing a sense of the scope of potential unmet treatment needs in the general population; and (v) to evaluate PTSD diagnosis in preadolescence in relation to psychiatric treatment utilization.

## Methods

### Sample and Procedure

Data for the current study were drawn from the Adolescent Brain and Cognitive Development (ABCD) dataset (release 5.0), a national study of adolescent brain development. Participants (unweighted *n* = 11,875) were recruited from 21 catchment sites across the United States. These catchment sites covered over 20% of the recruited population and the total population encompassed by these sites reasonably matched the sociodemographics of the U.S. population as a whole, but with certain groups (e.g., African American children) oversampled to ensure that site locations would not bias the sample and that the sample would reflect the sociodemographic variations of the U.S. population. Complete details about the sampling strategy and design, as well as weighting procedures, have previously been reported.^7^

Children and parents were recruited and assessed when children were between 9 and 10 years old (at time of assessment, weighted *M* = 9.50 years old, SE = 0.01 years). Measures and procedures were standardized across recruitment sites. For parents, measures were a combination of computerized assessments about themselves, the family, and their child, and the children were given computerized assessments about themselves.^8^

### Measures

#### Sociodemographic characteristics.

The following sociodemographic information was obtained from the parents: child sex, ethnicity (Hispanic or non-Hispanic), and race (Black, white, multiracial, American Indian/Alaskan Native, Asian, and other). Due to the small number of unweighted cases of PTSD for the American Indian/Alaskan Native and Asian racial groups, categories for race were collapsed into “Black,” “white,” “multiracial” and “other.” Parents also reported their education (collapsed into less than high school, high school or GED, some college, and college graduate), and parent marital status (collapsed into married and other). Sexual orientation was obtained from the children through a question asking if they were gay or bisexual, to which they could respond “yes,” “maybe,” “no,” or “I do not understand this question.” Responses of “yes” and “maybe” were combined to create a “gay, bisexual, or questioning” category, while responses of “no” formed a heterosexual category, and a third category was created of participants who did not understand the question. Family economic insecurity was calculated by summing the answers to seven questions regarding ability to pay for needed services (i.e., food, phone bill, rent or mortgage, eviction, utilities bill, doctors’ and dentists’ visits). This measure captures relative deprivation, a more sensitive indicator of the impact of economic circumstances than annual income.^9^ Higher scores indicated greater economic insecurity.

#### Psychiatric diagnoses and treatment utilization.

Lifetime and current diagnoses were determined using the youth version of the Kiddie Schedule for Affective Disorders and Schizophrenia-Present and Lifetime Version for DSM-5 (K-SADS-PL).^10,11^ Children and parents separately reported on child’s symptoms and behaviors for current (i.e., past 2 weeks) and past (i.e., prior to the past 2 weeks) psychopathology. Following standard clinical practice, a child was determined to have a psychiatric diagnosis if they met criteria based on parent report, child report, or both.^5^ In addition to PTSD, diagnoses included in the present study were psychotic disorder, major depressive disorder, separation anxiety, social anxiety disorder, specific phobia, generalized anxiety disorder, conduct disorder, oppositional defiant disorder, obsessive compulsive disorder, and eating disorders (i.e., anorexia nervosa, bulimia nervosa, and binge-eating disorder). Parents were presented with a standardized list of traumatic experiences and asked if their child had experienced any of the events. A trauma type count variable was calculated from the number of trauma types endorsed. In the K-SADS-PL, parents were asked if their child had ever received mental health treatment in their lifetime across a variety of modalities and treatment settings (e.g., psychotherapy, medication).

### Statistical Analyses

Analyses were conducted in Statistical Package for the Social Sciences (SPSS; version 25.0.0.0), and data were weighted using propensity weights to generate population-representative estimates. Cross-tabulations were calculated to estimate current and lifetime prevalence of PTSD, as well as lifetime prevalence of PTSD for each sociodemographic factor and psychiatric diagnosis. Associations of PTSD with sociodemographic factors were analyzed first with a series of univariate logistic regression analyses with lifetime PTSD as the criterion variable, followed by a multivariate regression analysis with all sociodemographic factors and number of traumatic events experienced included. Similarly, a series of univariate logistic regression analyses was conducted with lifetime psychiatric diagnoses as predictor variables and lifetime PTSD as the criterion variable. A multivariate logistic regression model was then generated with all psychiatric disorders included in the analysis, and child sex, sexual orientation, ethnicity, race, family economic insecurity, and traumatic event count entered as covariates. These sociodemographics were chosen as covariates based on previous literature providing evidence for their association with PTSD.^3,12–15^ Similarly, a bivariate logistic regression model was created with number of lifetime psychiatric disorders, excluding PTSD, as a predictor of lifetime PTSD. This was followed with a multivariate model that included child sex, sexual orientation, ethnicity, race, family economic insecurity, and traumatic event count as covariates.

Next, to evaluate the clinical sequelae of past PTSD, a bivariate regression analysis was conducted with past (i.e., prior to the past 2 weeks) PTSD as the predictor of interest and current first onset (i.e., past 2 weeks) of any psychiatric diagnosis (excluding PTSD) as the outcome variable. A multivariate logistic regression model was then generated with all past psychiatric disorders included in the analyses. Static sociodemographic characteristics (i.e., child sex, race, and ethnicity) were entered as covariates so as to maintain clean temporal separation between all predictors and the outcome variable. Additionally, to provide a stringent evaluation of the clinical sequelae of PTSD, we excluded from these analyses any children with past PTSD who still met diagnostic criteria for this disorder in the past 2 weeks, thus allowing a determination of whether PTSD in preadolescence is associated with negative clinical outcomes even after no longer meeting criteria for PTSD.

Cross-tabulations were then used to estimate lifetime prevalence of psychiatric treatment utilization among children with lifetime PTSD. Finally, a bivariate logistic regression analysis was conducted with lifetime PTSD as a predictor variable and utilization of any mental health services as the criterion variable, followed by a multivariate logistic regression analysis predicting utilization of any mental health services with PTSD as the predictor of interest, and with all other psychiatric diagnoses, child sex, sexual orientation, ethnicity, race, family economic insecurity, and traumatic event count as covariates.

Coefficients were exponentiated to create odds ratios (ORs) with 95% confidence intervals (CIs), and interpreted based on effect sizes following standard convention.^16^ Statistical significance was set as *p* < .05 using a two-tailed test. Multiple comparisons in univariable analyses were corrected using the Benjamini-Hochberg procedure.

## Results

### Prevalence and sociodemographic correlates

Lifetime prevalence of PTSD in the current sample was 2.17% (SE = 0.16), and 2-week prevalence was 0.12% (SE = 0.04). In bivariate analyses (Table 1), no differences in lifetime prevalence of PTSD were found for sex or ethnicity. However, higher odds of PTSD were found for children who identified as gay, lesbian, or questioning, and children who were black or multiracial. Among parental factors, having parents who had completed some college and were not married were associated with higher odds of PTSD, as was family economic insecurity. When all sociodemographic variables were considered together with count of traumatic events experienced in a multivariate analysis, sexual minority status, having unmarried parents, and family economic insecurity remained significant predictors of lifetime PTSD with small to medium-to-large effects.

### Psychiatric diagnostic correlates

Lifetime prevalence, unadjusted ORs, and ORs adjusted for all sociodemographic factors and traumatic event count were calculated for each psychiatric disorder (Table 2). Lifetime prevalence of PTSD for each psychiatric diagnosis varied, with specific phobia having the lowest prevalence of comorbid PTSD at 4.79% (SE = 0.45) and psychosis having the highest prevalence at 14.44% (SE = 3.23).

Unadjusted ORs for each disorder were significant with medium to large effect sizes, with the odds of PTSD ranging from OR = 4.21 (95% CI = 3.13–5.66) for specific phobia to OR = 12.13 (95% CI = 9.00–16.35) for separation anxiety. In the multivariate analysis that included all psychiatric disorders, sociodemographic factors (child sex, sexual orientation, race, ethnicity, family economic insecurity), and trauma event count, reduced odds were observed across all disorders, but PTSD continued to be significantly predicted by separation anxiety, specific phobia, generalized anxiety disorder, oppositional defiant disorder, and obsessive compulsive disorder. Among the psychiatric disorders that remained significant predictors of PTSD in the multivariate model, the strength of the association ranged from a small effect of OR = 1.67 (95% CI = 1.14–2.42) for specific phobia to a medium-to-large effect of OR = 3.60 (95% CI = 2.37–5.46) for separation anxiety. The high comorbidity between PTSD and psychiatric correlates was highlighted in a regression analysis finding a positive association between number of lifetime psychiatric disorders and lifetime PTSD, a finding that held in a multivariate analysis that included child sex, sexual orientation, race, ethnicity, family economic insecurity, and traumatic event count as covariates.

### Clinical sequelae of PTSD

Analyses were conducted to further examine the relationship between PTSD and subsequent first lifetime onset of other psychiatric disorders (Table 3). In a bivariate model, past PTSD predicted greater odds of current (i.e., past 2 weeks) first onset of a new psychiatric disorder (excluding PTSD). After other past psychiatric diagnoses and static demographic characteristics (i.e., sex, race, and ethnicity) were accounted for, PTSD continued to be positively predictive of current first onset of a new psychiatric disorder, with an effect size in the small-to-medium range.

### Treatment utilization

Among the children who had a lifetime PTSD diagnosis, 63.0% (SE = 3.5) received at least one type of mental health treatment in their lifetime. In a bivariate logistic regression analysis, lifetime PTSD diagnosis predicted greater odds of engaging with any mental health services (OR = 9.36, 95% CI = 6.91–12.68, *p* < .001). Further, PTSD remained a significant predictor of mental health services engagement even after accounting for comorbid psychiatric diagnoses, child sex, sexual orientation, race, ethnicity, family economic insecurity, and traumatic event count, with an effect size in the medium range (OR = 2.16, 95% CI = 1.43–3.24, *p* < .001).

## Discussion

The current study presents the most comprehensive analysis to date of preadolescent PTSD. It evaluated the prevalence of PTSD, sociodemographic correlates, associations with other psychiatric diagnoses, and psychiatric treatment utilization in a population-based sample. When considered with lifetime prevalence estimates for PTSD from epidemiological studies with adolescents (4.7%)^3^ and adults (7.3–7.8%),^2,13,15^ the lifetime prevalence in the current study (2.23%) fits within a pattern of increasing prevalence of PTSD across development.

Among sociodemographic factors associated with PTSD, sexual minority status had the largest effect, and the lifetime prevalence of PTSD in this group was nearly three times that for the general population. This is a critical finding, as sexual orientation has been predominantly studied in relation to mental health outcomes in adolescence and adulthood. Indeed, it has not been previously assessed in relation to PTSD in individuals younger than 12,^17^ despite research showing differences in sexual orientation begin to emerge at age eight.^18^ This finding suggests that sexual minority individuals in the age group potentially most vulnerable to PTSD are also the least studied. Our findings therefore highlight the need for greater research on PTSD and potential processes relevant to risk and resilience in preadolescent sexual minority children.

Another notable sociodemographic factor associated with lifetime PTSD was family economic insecurity. This finding is consistent with prior literature.^19,20^ In particular, for adolescents, living in a low socioeconomic status (SES) environment has been associated with higher levels of PTSD.^21^ Further, within a sample of children and adolescents exposed to trauma, those who came from lower SES had higher rates of PTSD when compared to those who came from higher SES, suggesting that SES contributes unique risk over and above that of the traumatic experience.^20^ Additionally, adolescents from backgrounds of higher economic insecurity have been found to have poorer recovery from PTSD,^3^ which may relate in part to research on PTSD which has found limited finances and access to transportation to be barriers to accessing care.^22^ In sum, those who have high economic insecurity are not only at risk for PTSD, they also may be least likely to have the resources to mitigate risk and to access treatment.

The absence of a sex difference in PTSD in the present study is notable as it contrasts with epidemiological studies with adolescents^3^ and adults,^2^ as well as meta-analytical work^23^ which found greater prevalence of PTSD among females. However, risk for PTSD associated with being females increases with age.^14^ Thus, our findings suggest that sex differences in risk for PTSD are not yet apparent in preadolescence, and instead emerge in adolescence. Identifying determinants of this emergence of sex difference in PTSD in adolescence is important for informing risk identification and prevention strategies.

PTSD was associated with most other psychiatric disorders in the multivariate analysis even when accounting for prior trauma exposure, with separation anxiety emerging as the single strongest diagnostic predictor of PTSD. This pattern of high psychiatric comorbidity is consistent with findings from population-based studies with adults.^2,24^ Further, the relative non-specificity in correlated psychiatric disorders speaks to the challenge of identifying risk for PTSD based on presence of other psychiatric diagnoses alone and that identifying underlying transdiagnostic indices of risk may be a fruitful avenue for future investigation.

A notable finding was the low 2-week prevalence of PTSD (0.12%) relative to lifetime prevalence (2.17%), suggesting that PTSD tends not to follow a chronic course during preadolescence. This differs from the finding that this disorder often follows a chronic course in adults, with a median time to remission of 36 months among those who received treatment in this age group and 64 months among those who do not, and furthermore, over a third of adults do not experience remission even after many years, regardless of receiving treatment.^2^ PTSD has similarly been found to follow a persistent course in adolescents.^6^ We caution against interpreting the contrastingly high remission rate for PTSD in the current study as indicative that PTSD is less of a clinical concern in preadolescents because of its tendency to remit over relatively brief periods of time. Rather, our finding that a past history of PTSD, even after remission is achieved, temporally predicts first lifetime onset of other psychiatric disorders suggests that some measure of clinical risk persists past diagnostic remission and thus that a prior history PTSD may serve as an important marker of risk for future mental health problems.

A significant proportion of preadolescents with PTSD (37.0%) did not receive any mental health treatment. This is concerning given the aforementioned findings of risk for new psychiatric disorder onset associated with past PTSD and because untreated PTSD in youth can lead to increased healthcare costs, decreased performance in school, and lower rates of high school graduation.^25^ Research has shown that these impairments carry into functional impairments in young adulthood, including isolation, social loneliness and not working or in school.^15^ Therefore, reducing untreated PTSD in preadolescence is critical to reduce its potential long-term negative outcomes. Future research should identify factors associated with unmet treatment needs in this population, as well as strategies to address these needs.

Limitations of the current study should be noted. First, it was not possible to examine sociodemographic correlates and psychiatric comorbidity for current PTSD due to its low prevalence. Accurately characterizing correlates of current PTSD is critical for informing screening and intervention strategies. The importance of this lies in part in the aforementioned observation that most children with lifetime PTSD no longer met diagnostic criteria at the time of assessment, meaning that caution should be taken in assuming correlates of lifetime PTSD generalize to current PTSD in this population. This limitation notwithstanding, the current findings regarding lifetime PTSD are of clinical importance given the significant psychiatric sequelae of this disorder even after diagnostic remission is achieved.

This study was also limited to cross-sectional and retrospective temporal analyses. Although concerns regarding accurate retrospective recall of psychiatric diagnoses^26^ is reduced given the age of the population, and it was possible to achieve clean temporal separation in analyses of clinical sequelae of PTSD, prospective longitudinal analyses are important for their potential to provide unique insight into the phenomenology of PTSD in this population. Given the high remission of PTSD in this age, for example, a prospective design is necessary to observe sufficient new onsets of PTSD to evaluate sociodemographic and clinical characteristics temporally preceding its occurrence. Additionally, to our knowledge, our work is one of the first to study sexual orientation as a predictor of PTSD in preadolescents, and found that identifying as a sexual minority youth connoted greater odds of developing PTSD compared to identifying as heterosexual. Given the strength of this association, further research is needed to delineate the mechanisms underlying risk for this population (e.g., within minority stress frameworks).^27^

## Conclusion

Overall, the current findings reveal that although most children with PTSD no longer meet diagnostic criteria with time, it is nonetheless associated with significant negative long-term clinical sequelae, even after PTSD no longer meets diagnostic criteria, making the high proportion of untreated PTSD in this population a particular clinical concern. In contrast to the case of adolescents and adults, with PTSD more prevalent among females, our finding of the absence of a sex difference in PTSD prevalence suggests that comparable weight should be given to both males and females in identifying risk of PTSD in preadolescents.

## Figures and Tables

**Table 1:** Associations between sociodemographic factors and lifetime posttraumatic stress disorder (unweighted n = 11,690).

Predictor	% (SE)	Univariate^a^		Multivariate^b^	
		Odds Ratio (95% CI)	*p*	Odds Ratio (95% CI)	*p*
*Sex*					
Female	2.02 (0.22)	0.87 (0.65–1.17)	.36	0.90 (0.65–1.25)	.53
Male	2.31 (0.22)	1.00		1.00	
*Sexual orientation*					
Gay, lesbian, or questioning	6.47 (2.33)	3.11 (1.44–6.73)	< .01	2.87 (1.41–5.86)	< .01
Did not understand question	1.92 (0.30)	0.88 (0.61–1.26)	.48	1.13 (0.76–1.67)	.56
Heterosexual	2.18 (0.18)	1.00		1.00	
*Race*					
Black	2.87 (0.42)	1.55 (1.08–2.20)	.02	0.75 (0.48–1.18)	.22
Multiracial	4.12 (0.67)	2.24 (1.52–3.30)	< .001	1.54 (0.98–2.41)	.06
Other	1.87 (0.54)	1.00 (0.54–1.83)	.99	1.17 (0.60–2.28)	.64
White	1.88 (0.19)	1.00		1.00	
*Ethnicity*					
Hispanic	2.00 (0.32)	0.91 (0.64–1.30)	.61	0.79 (0.52–1.21)	.29
Not Hispanic	2.19 (0.18)	1.00		1.00	
*Parental education*					
< High school	2.61 (1.38)	1.42 (0.48–4.18)	.53	1.48 (0.52–4.27)	.46
High school or GED	2.42 (0.39)	1.31 (0.90–1.92)	.16	0.95 (0.61–1.46)	.81
Some college	2.92 (0.43)	1.59 (1.11–2.28)	.01	0.96 (0.63–1.47)	.87
College graduate	1.85 (0.18)	1.00		1.00	
*Parent marital status*					
Other	3.82 (0.34)	3.51 (2.59–4.75)	< .001	2.82 (1.85–4.31)	< .001
Married	1.12 (0.14)	1.00		1.00	
Family economic insecurity index*	—	1.52 (1.42–1.62)	< .001	1.35 (1.21–1.49)	< .001

*Note*. Weighted prevalence of PTSD is presented for each predictor. CI = confidence interval; GED = general education development

* Weighted prevalence of PTSD was not calculated for family economic insecurity index as it is not a categorical variable.

a All analyses remained significant after applying Benjamini-Hochberg corrections.

b The multivariate model includes all sociodemographics, and count of traumatic experiences as a covariate.

**Table 2:** Associations between psychiatric disorders and lifetime posttraumatic stress disorder (unweighted n = 11,690).

Predictor	% (SE)	Univariate^a^		Multivariate^b^	
		Odds Ratio (95% CI)	*p*	Odds Ratio (95% CI)	*p*
Number of disorders^c^	—	2.30 (2.14–2.48)	< .001	2.07 (1.90–2.26)	< .001
Psychosis	14.44 (3.23)	8.18 (4.79–13.96)	< .001	1.81 (0.85–3.86)	.13
Major depressive disorder	8.60 (1.26)	5.20 (3.65–7.40)	< .001	1.56 (0.98–2.48)	.06
Separation anxiety	12.27 (1.17)	12.13 (9.00–16.35)	< .001	3.60 (2.37–5.46)	< .001
Social anxiety	7.58 (1.17)	4.33 (3.00–6.23)	< .001	1.05 (0.63–1.74)	.86
Specific phobia	4.79 (0.45)	4.21 (3.13–5.66)	< .001	1.66 (1.14–2.42)	< .01
Generalized anxiety disorder	14.33 (1.64)	10.92 (7.94–15.01)	< .001	2.71 (1.68–4.38)	< .001
Conduct disorder	11.38 (1.90)	6.82 (4.56–10.19)	< .001	1.24 (0.69–2.21)	.48
Oppositional defiant disorder	8.57 (0.81)	8.40 (6.25–11.30)	< .001	3.02 (2.03–4.49)	< .001
Eating disorder	10.54 (2.99)	5.51 (2.90–10.45)	< .001	0.97 (0.40–2.37)	.94
Obsessive compulsive disorder	8.12 (0.90)	5.70 (4.22–7.71)	< .001	1.97 (1.31–2.95)	.001

*Note*. Weighted prevalence of PTSD is presented for each predictor. Horizontal lines serve to demarcate separate models. CI = confidence interval

a All analyses remained significant after applying Benjamini-Hochberg corrections.

b The multivariate models include child sex, sexual orientation, ethnicity, race, family economic insecurity, and count of traumatic experiences as covariates. The multivariate analysis evaluating individual disorders as predictors of PTSD also included all 10 disorders and count of traumatic experiences in the model.

c Analyses of number of disorders as predictors of PTSD excluded PTSD from the predictor variable.

**Table 3:** Past posttraumatic stress disorder temporally predicting current onset of new psychiatric disorders (unweighted n = 11,677).^a^

Predictor	Odds Ratio (95% CI)	*p*
*Bivariate model*		
Post-traumatic stress disorder	4.98 (3.66–6.69)	< .001
*Multivariate model* ^b^		
Post-traumatic stress disorder	2.05 (1.43–2.93)	< .001
Psychosis	1.48 (0.65–3.34)	.35
Major depressive disorder	1.99 (1.55–2.55)	< .001
Separation anxiety	2.30 (1.90–2.78)	< .001
Social anxiety	1.60 (1.25–2.05)	< .001
Specific phobia	1.16 (1.01–1.34)	.04
Generalized anxiety disorder	1.96 (1.51–2.56)	< .001
Conduct disorder	3.94 (2.58–6.01)	< .001
Oppositional defiant disorder	1.07 (0.86–1.31)	.56
Eating disorder	2.60 (1.22–5.55)	.01
Obsessive compulsive disorder	0.94 (0.70–1.26)	.65

*Note*. CI = confidence interval

a Participants with current PTSD were excluded from these analyses.

b In addition to all past diagnoses, only static demographic characteristics (i.e., child sex, race, and ethnicity) were included as covariates to maintain clean temporal separation between predictors and current onset of new psychiatric disorders.
